# Overexpression of Bovine FcRn in Mice Enhances T-Dependent Immune Responses by Amplifying T Helper Cell Frequency and Germinal Center Enlargement in the Spleen

**DOI:** 10.3389/fimmu.2015.00357

**Published:** 2015-07-20

**Authors:** Zita Schneider, Péter Károly Jani, Bence Szikora, Attila Végh, Dorottya Kövesdi, Attila Iliás, Judit Cervenak, Péter Balogh, István Kurucz, Imre Kacskovics

**Affiliations:** ^1^Department of Immunology, Eötvös Loránd University, Budapest, Hungary; ^2^ImmunoGenes Kft, Budakeszi, Hungary; ^3^Department of Immunology and Biotechnology, Medical School, University of Pécs, Pécs, Hungary; ^4^Lymphoid Organogenesis Research Group, Szentágothai Research Center, University of Pécs, Pécs, Hungary; ^5^ImmunoGenes-ABS Zrt, Budapest, Hungary

**Keywords:** FcRn overexpression, germinal center, humoral immune response, antigen presentation

## Abstract

The neonatal Fc receptor (FcRn) plays key roles in IgG and albumin homeostasis, maternal IgG transport, and antigen presentation of IgG-opsonized antigens. Previously, we reported that transgenic (Tg) mice that overexpress the bovine FcRn (bFcRn) have augmented T-dependent humoral immune response with increased IgG protection, higher level of antigen-specific antibodies, greater number of antigen-specific B cells, and effective immune response even against weakly immunogenic epitopes. In the current study, we analyzed the localization of the bFcRn in secondary lymphoid organs, and focused to demonstrate the *in vivo* impact of its overexpression in the spleen on the course of antibody production. bFcRn was highly expressed by red pulp macrophages and marginal zone macrophages in the spleen and by subcapsular sinus macrophages and macrophage-like cells in the interfollicular areas in the lymph node cortex. We also demonstrated that splenic dendritic cells of Tg mice express bFcRn and intraperitoneal immunization of these mice with T-dependent antigens led to more than threefold increase in the number of antigen-specific activated T helper cells with increased size and numbers of germinal centers compared to wild-type controls. bFcRn expression in splenic B cells was also detected and that may also contribute to the enhanced B cell activation. Finally, we demonstrated that these Tg mice developed efficient immune response against very low dose of antigen, reflecting another important practical benefit of these Tg mice.

## Introduction

Based on a recent survey, wild-type (wt) mice are the source of two-third of all late stage and marketed therapeutic monoclonal antibodies (mAbs), primarily in the form of humanized antibodies (1). In recent years, there has been an increasing demand for the development of faster and more efficient technologies for the production of high-affinity mAbs against specific epitopes of targets that are considered to be weakly immunogenic or even tolerogenic. Therefore, it is important to increase the efficiency of the humoral immune response of the mouse to enhance their capability to produce hybridomas against challenging targets.

Although the neonatal Fc receptor (FcRn) was originally identified as the protein that mediates the maternal immune transport (2, 3), it was soon realized that this receptor is a key player in regulating the transport of IgG within and across cells of diverse origins. Furthermore, FcRn also serves to rescue IgG and albumin in capillary endothelial and hematopoietic cells from degradation, prolonging their half-lives (4). More recently, FcRn was demonstrated to play major roles in antigen (Ag) presentation in case of Ag–IgG immune complexes (IC) by professional Ag-presenting cells (APCs) stimulating MHC class II and also MHC class I-related T cell activation (5).

We have previously shown that overexpression of the FcRn in transgenic mice and rabbits extended IgG half-life (6, 7). These animals develop higher Ag-specific antibody titers, which is not merely the result of the better IgG protection but primarily due to the increased number of activated Ag-specific B cells, upon immunization on the course of T-dependent immune response (8–10). We have been able to take advantage of the enhanced humoral immune response of these animals by developing high quality antibodies against challenging antigens, like against the conserved segment of the influenza hemagglutinin subunit 2 or the GPCR cannabinoid receptor type 1 (11, 12).

In support of the role of FcRn in Ag presentation in mouse and human, we previously demonstrated that the bovine FcRn (bFcRn) is expressed in peritoneal macrophages (MΦ) and bone marrow-derived dendritic cells in bFcRn Tg mice (13). We also confirmed that the Tg bone marrow-derived dendritic cells stimulate T helper cells significantly more efficiently when loaded with Ag–IgG ICs than those derived from wt mice (13). These *in vitro* data suggested that overexpression of the bFcRn in APCs results in augmented Ag presentation leading to increased number of Ag-specific B cells in the bFcRn Tg mice. Yet, the augmented antigen presentation has not been analyzed in the secondary lymphoid organs of these Tg mice, and thus the relationship between bFcRn overexpression and the increased number of antigen-specific B cells during the humoral immune response still had to be confirmed.

Therefore, we first analyzed the architecture of the spleen and identified the bFcRn-positive cells in the secondary lymphoid organs of the bFcRn Tg mice comparing them to the expression pattern of the mouse FcRn (mFcRn) that had been reported for phagocytic cells and APCs (14–18). This issue is important as the bFcRn expression is regulated by its own regulatory elements in these Tg mice (6) that are similar to its mouse and human counterparts with some differences. Notably, we found upregulation of the bFcRn by NFκB in the spleen and peritoneal MΦs (19) similarly to the human FcRn (hFcRn) (20), while this has not been reported for the mFcRn (21). Despite the difference of cytokine inducibility, mFcRn and hFcRn are considered to be expressed in the same cell types and thus mouse is a well-established model for analyzing the distribution and function of the hFcRn. Nevertheless, there is at least one exception from this general concept as mFcRn is expressed only in newborn enterocytes, while hFcRn is expressed in these cells throughout life (4), suggesting careful analysis of the expression and function of the transgenic bFcRn in these Tg mice is salutary.

We also analyzed the Ag presentation efficiency of the splenic APCs by immunizing Tg and wt mice with ovalbumin (OVA) and studied recall activation of T cells from unsorted splenocytes of these mice. Then, we determined germinal center (GC) size distributions and kinetics of their formation in the spleen followed by trinitrophenyl (TNP)-KLH immunization. Finally, the efficiency of the Ag-specific IgG antibody responses against low-dose antigen was evaluated.

## Materials and Methods

### Animals

We used hemizygous transgenic mice that carry five copies of the bFcRn α-chain encoding gene (bovine FCGRT) in addition to the endogenous mouse FCGRT gene on BALB/c genetic background [BALB/c_Tg5_bFCGRT(19); 19 refers to the founder line] that we have previously generated (9). Wt BALB/c mice were littermates of the transgenic animals resulted from hemizygous breeding. Mice were kept under specified pathogen free (SPF) conditions in individual ventilation cages (IVC) in the animal house of the Department of Immunology, Eötvös Loránd University, Budapest, Hungary. Treatments of mice in this study were carried out in strict accordance with the recommendations in the Guide of the Institutional Animal Care and Ethics Committee at Eötvös Loránd University that operated in accordance with permissions 22.1/828/003/2007 and XIV-I-001/517-4/2012 issued by the Food Chain Safety and Animal Health Directorate of the Government Office of Pest County, Hungary.

### Immunohistochemical localization of the bovine and mouse FcRn in the spleen

Ten-week-old female bFcRn transgenic and wt BALB/c mice were immunized intraperitoneally (i.p.) with 50 μg keyhole limpet hemocyanin (KLH; Sigma-Aldrich) conjugated to TNP in complete Freund adjuvant (CFA), boosted 3 weeks later with 25 μg TNP-KLH in incomplete Freund adjuvant (IFA), and euthanized 3 days thereafter. Spleens of non-immunized and immunized mice were frozen in liquid N_2_, embedded in Killik cryostat embedding medium (Bio-Optica, Milano, Italy), and 10–15 μm sections were made and fixed with acetone. Before staining, sections were blocked by 5% BSA for 20 min at RT. Cell populations were identified with Alexa Fluor 568-labeled anti-Thy-1 (clone IBL-1), Cy3-labeled anti-MARCO (clone IBL-12), and MOMA (sialoadhesin/CD169, clone IBL-13) mAbs, that were developed in our laboratory (22–24). Anti-B220 (clone RA3-6B2), F4/80, Gr-1/Ly-6C/G (clone RB6-8C5), MAdCAM-1 (clone MECA-367) were obtained from the American Type Culture Collection, Rockville, MD, USA. FITC-labeled and PE-conjugated goat anti-rat Igs were purchased from BD Pharmingen (San Diego, CA, USA). Dendritic cells were identified using hamster mAb against mouse CD11c (clone N418). Bovine FcRn-expressing cells were identified by Alexa Fluor 488- or Alexa Fluor 647-conjugated anti-bFcRn mAb (Z15_6D5/8) that was developed in our laboratory (19), while mFcRn expressing cells were stained by Armenian hamster anti-mFcRn serum provided by Dr. S. Akilesh (Washington University, St. Louis, MO, USA) (14) and FITC-labeled anti-hamster rabbit IgG (Southern Biotechnology Associates Inc., Birmingham, AL, USA). The pictures of fluorescence staining were edited with Adobe Photoshop, using adjustments for black level and hue for the entire images.

### *Ex vivo* antigen presentation assay

Ten-week-old female bFcRn transgenic and wt BALB/c mice were immunized i.p. with 100 μg OVA in CFA. Mice were boosted i.p. with 50 μg OVA in IFA on day 14, and sacrificed on day 21. Single-cell suspension was prepared from the spleen of the mice in RPMI medium containing 10% FCS (Sigma-Aldrich). To analyze the number of Ag-specific T helper cells after immunization, we used BD Mouse IL-2 ELISPOT Set (BD Biosciences, NJ, USA) according to the manufacturer’s instructions with slight modifications. Ninety-six-well ELISPOT plates were coated with 5 μg/ml purified anti-mouse IL-2 antibody in 100 μl PBS and incubated at RT for 2 h. Plates were washed with PBS and blocked with RPMI + 10% FCS for 1 h at RT. Spleen cells were plated in concentrations of 1 × 10^6^ and 5 × 10^5^ cells per well in 200 μl of RPMI + 10% FCS. Cells were pulsed with OVA or KLH at 10 and 50 μg/ml final concentration, which was determined in pilot experiments. For negative control, splenocytes were plated without Ag-restimulation. Plates were incubated for 22 h at 37°C in 5% CO_2_ atmosphere then washed thoroughly with PBS-Tween (0.05%), and biotinylated goat anti-mouse IL-2 antibody was added at 2 μg/ml concentration in 100 μl PBS-Tween and incubated for 2 h at room temperature. After washing, streptavidin-HRP conjugate was added in 100 μl PBS-Tween. After 1 h incubation at RT, plates were washed five times with PBS-Tween and then five times with PBS. The plates were then incubated with 3-amino-9-ethylcarbazole (AEC, Sigma-Aldrich), and H_2_O_2_ at room temperature in 0.1M acetate buffer, pH 5.2, and the reaction was terminated by a wash with distilled water. The spots were counted in the ImmunoScan ELISPOT reader (Cellular Technology) and evaluated by ImmunoSpot software version 3.2 (Cellular Technology).

### Flow cytometry

Single-cell suspensions from spleens were made and 1 × 10^6^ isolated cells were stained with fluorochrome-conjugated specific antibodies at 4°C for 50 min in staining buffer (PBS with 0.1% BSA and 0.1% sodium azide), washed twice, and then analyzed using a FACSCalibur equipped with CellQuest software (BD Biosciences, San Jose, CA, USA). Anti-mouse CD45R/B220-PECy5 or CD45R/B220-PerCP-Cy5.5, CD19-FITC, CD3-PE, CD11b-PerCP-Cy5.5 were obtained from eBioscience (San Diego, CA, USA). Anti-mouse CD4-PE, CD11c-PE, Gr-1 (Ly-6G)-PE were purchased from BD Pharmingen (San Diego, CA, USA) and anti-mouse F4/80-Alexa Fluor 647 is from Caltag Laboratories (Buckingham, UK). Isotype controls were obtained from BD Pharmingen or eBioscience.

For intracellular staining, cells were incubated with fluorochrome-conjugated antibodies specific for cell surface molecules, as described previously, then washed twice, and fixed by adding 4% formaldehyde for 10 min at 4°C. Subsequently, fixed cells were treated with 0.2% saponin (Sigma-Aldrich) and 0.1% BSA (Sigma-Aldrich) containing PBS for 10 min, washed, and stained with Alexa Fluor 488- or Alexa Fluor 647-conjugated anti-bFcRn mAb (Z15_6D5/8) in the same saponin-containing buffer for 1 h at 4°C. After staining, cells were washed twice and all samples were resuspended in staining buffer and analyzed by FACS Aria III equipped with FACSDiva software (BD Biosciences, San Jose, CA, USA). Data were evaluated with FlowJo software (FlowJo LLC, Ashland, OR, USA).

### Lectin-immunohistochemical analysis of the number, size, and kinetics of spleen germinal centers

Ten-week-old female bFcRn transgenic and wt BALB/c mice were immunized i.p. with 50 μg TNP-KLH in CFA, boosted 3 weeks later with 25 μg TNP-KLH in IFA, and were analyzed on days 24, 28, and 42 of the immune response (3 Tg and wt mice in each group). Two independent sections were made for each spleen specimen (S1 and S2, distance at least 500 μm) for two-dimensional cross-sectional evaluation of GCs. Sections were mounted onto ready-to-use microscope slides (Menzel, Thermo Scientific, MA, USA), were air-dried overnight, fixed in cold acetone for 5 min, air-dried again, and stored at −80°C until analysis. Anti-mouse IgD (BD Pharmingen, Franklin Lakes, NJ, USA) and biotinylated peanut agglutinin (PNA; Sigma-Aldrich) were used to analyze GC in the spleen. Developing rat anti-mouse IgD and PNA double labeling was done either by using enzymatic detection in conjunction with anti-rat IgG-HRPO conjugate (using H_2_O_2_ and AEC substrate) and extravidin-AP (developed with NBT-BCIP substrate combination) or fluorescent detection using PromoFluor350 labeled streptavidin (PromoKine, Germany) and anti-rat IgG-FITC (BD Pharmingen). Digital images of GCs, as identified by PNA within IgD positive mantle zone, were acquired on an Olympus BX61 microscope using a 10× objective and the analySIS software (Olympus, Germany). GC boundaries were manually assigned to each GC, and area was determined using analySIS software.

### Immune response against low amount of Ag

Trinitrophenyl-KLH was used for immunization, and TNP-BSA was used as a test Ag for determining anti-hapten antibody level, as published earlier (9). The number of incorporated TNP residues per KLH molecule was 400 and per BSA molecule was 24. Animals were i.p. immunized with different amount of TNP-KLH in CFA and challenged 21 and 42 days later with TNP-KLH in IFA according to Table 1. High-binding ELISA plates (Costar 9018, Corning, NY, USA) were coated with TNP-BSA (4 μg/ml); in 0.1M sodium carbonate–bicarbonate buffer (pH 9.6) for 2 h at room temperature and then were washed with 0.1M PBS (pH 7.2) containing 0.05% Tween 20 (PBS-Tween) and blocked with PBS containing 1% BSA for 1 h at room temperature. Serially diluted serum samples were added to the wells and incubated for 1 h at room temperature. Each plate included standard controls of serially diluted Ag-specific immune sera. After washing, bound serum IgG was detected by HRP-labeled goat anti-mouse IgG (1:4000-fold dilution, Southern Biotechnology Associates, Birmingham, AL, USA). The peroxidase-conjugated antibodies were detected using tetramethylbenzidine (TMB, Sigma-Aldrich) as the chromogen and hydrogen peroxide as the substrate, and OD at 450 nm was measured with the Multiscan ELISA Plate Reader (Thermo EC). Ag-specific IgG titers as half-maximal values were determined by GraphPad Prism 5 non-linear regression to the hyperbolic saturation function.

**Table 1:** **Immunization protocols for testing the immune response to low-dose TNP-KLH**.

Cohorts *(5 BALB/c – 5 bFcRn Tg BALB/c)*	Immunization day	TNP-KLH (**μ**g/animal) i.p.	Adjuvant
1	0	100	CFA
	21	50	IFA
	42	50	IFA
2	0	10	CFA
	21	5	IFA
	42	5	IFA
3	0	0.5	CFA
	21	0.5	IFA
	42	0.5	IFA

### Statistical analysis

Statistical differences were assessed by Student’s two-tailed *t* test and two-way ANOVA. Values were considered significant and were indicated as follows: *P < 0.05; **P < 0.01; ***P < 0.001.

## Results

### bFcRn is expressed in the spleen and lymph node

**Figure 1:**
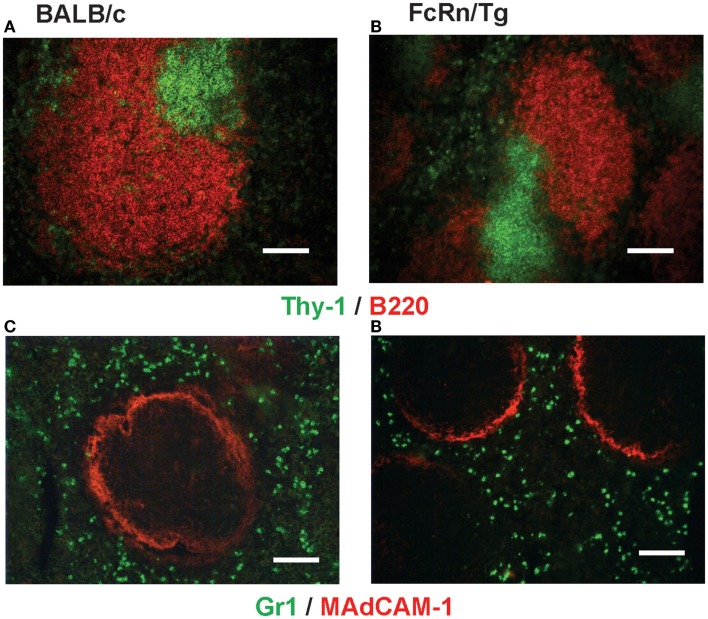
**Histological analysis of the spleen of bFcRn Tg mice**. White pulp in spleens in wt **(A,C)** and Tg mice **(B,D)** contains clearly defined T cells (Thy-1/CD90, green) and B cells (B220, red) forming separate compartments. The white pulp is encircled with MAdCAM-1 positive (red) marginal sinus, separating the white pulp from the red pulp containing Gr1-positive (green) myeloid cells (representative examples for a cohort *n* > 6; scale bar: 100 μm).

**Figure 2:**
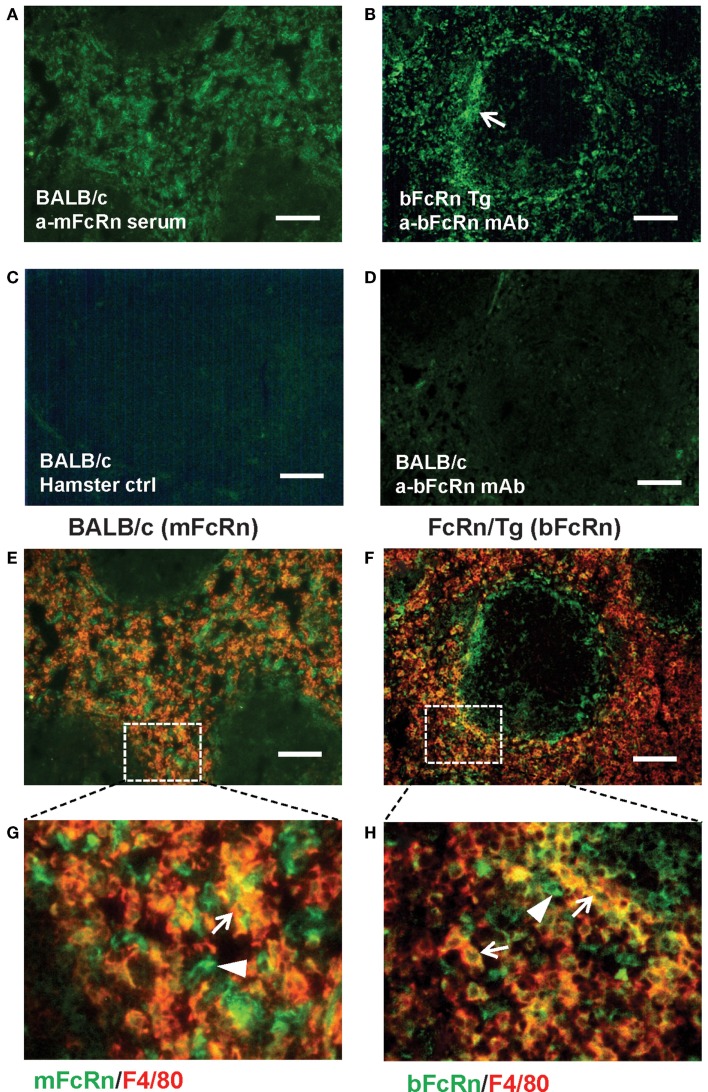
**Expression of FcRn and bFcRn in wt and bFcRn Tg mice in the red pulp**. mFcRn in wt mice was stained with anti-mFcRn or control hamster serum or anti-bFcRn mAb as indicated **(A,C,D)**. bFcRn in Tg samples **(B)** was reacted with mouse anti-bFcRn mAb conjugated to Alexa Fluor 488. Note the accentuated reactivity of bFcRn in the marginal zone region [**(B)**, arrow]. **(E,F)** illustrate co-expression of mFcRn and bFcRn with F4/80, with the insets **(G,H)** corresponding to the rectangles delineated with dotted line in **(E,F)** Arrows point to double-positive cells, arrowheads indicate FcRn-receptors only positive cells in **(G,H)** (representative examples for a cohort *n* > 6, bar size: 100 μm).

**Figure 3:**
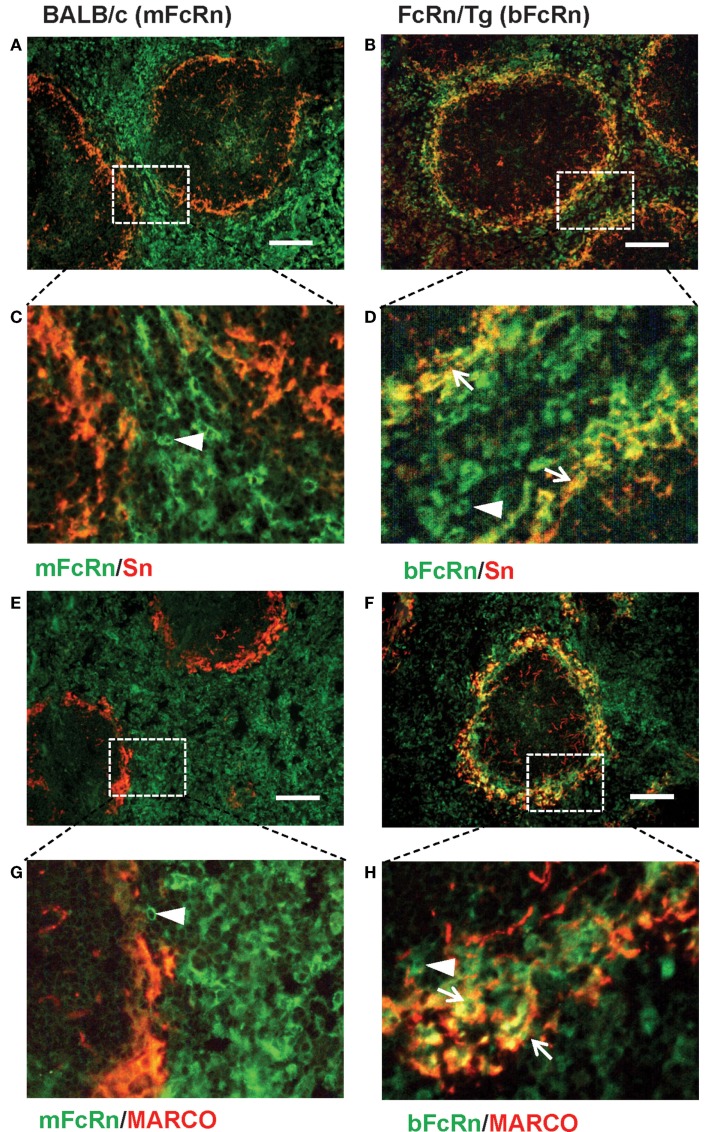
**Display of bFcRn by MZ macrophage subsets in Tg mice**. Co-labeling of spleen sections from wt mice for mFcRn **(A,C,E,G)** or from Tg mice for bFcRn **(B,D,F,H)** for sialoadhesin/CD169 **(A–D)** or MARCO **(E–H)** shows the lack of mFcRn expression in MZ, whereas in Tg mice, bFcRn is co-expressed by both MZ macrophage subsets. Arrows point to double-positive cells, arrowheads indicate FcRn-receptors only positive cells (representative examples for a cohort *n* > 6, bar size: 100 μm).

**Figure 4:**
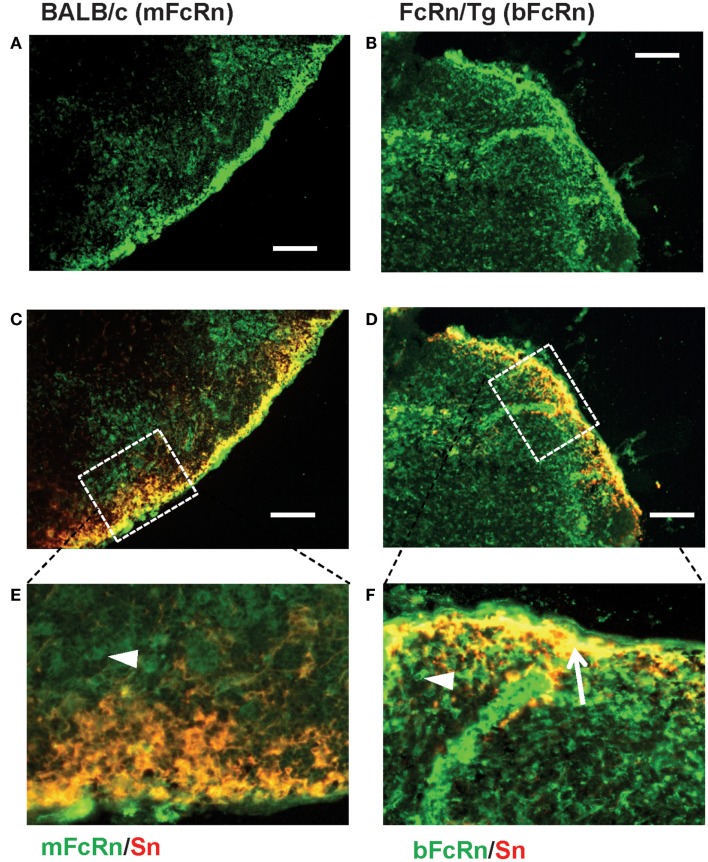
**Expression of FcRn in peripheral lymph nodes**. mFcRn in wt lymph nodes **(A,C,E)** and bFcRn in Tg lymph nodes **(B,D,F)** show slightly different tissue distribution, where wt mice express mFcRn (green) only in the interfollicular region, whereas in Tg mice, substantial co-expression of bFcRn (green) is seen with the Sn/CD169+ subcapsular metallophilic MΦs (red, overlap seen as yellow, indicated with an arrow pointing at a group of double-positive cells in **(F)**]. The high power insets **(E,F)** correspond to the dotted rectangular area in the merged pictures scale (representative examples for a cohort *n* > 6, bar size: 100 μm).

Transgenic mice that overexpress the bFcRn carry five copies of a bovine genomic fragment, encoding the bFcRn α-chain (FCGRT) (6). Upon immunization, these animals launch an enhanced antibody response against a variety of T-dependent antigens with mechanisms, which have not been completely elucidated yet. To test whether the expression of transgene containing all regulatory elements of the bFcRn gene or its chromosomal integration influences the architecture of spleen, we compared the structure of the wt and Tg spleen using tissue immunofluorescence for lymphoid compartmentalization and the expression pattern of endogenous mFcRn and transgenic bFcRn, respectively. We found that the spleen of Tg mice showed normal T-, B cell, and marginal zone (MZ) compartmentalization indistinguishable from wt controls using Thy-1, B220, Gr1, or MadCAM-1-specific labeling (Figure 1). Similar results were obtained with animals immunized with TNP-KLH (data not shown). Next, we correlated the cell-type-specific expression of bFcRn to the mFcRn in the spleen and lymph nodes. mFcRn localization was performed by using a mFcRn-specific hamster antiserum that had been developed and validated previously (14); as negative control, we used non-specific hamster serum with the same secondary reagent and found no specific staining. For bFcRn detection, we used the bFcRn-specific mAb (Z15_6D5/8) that we recently developed and showed that it does not cross-react with the endogenous mFcRn (19) (Figures 2A–D). Staining of F4/80-positive red pulp MΦs revealed an expression pattern of bFcRn in Tg mice, which is similar to the mFcRn patterns in wt mice (Figures 2E–H) and supporting previous studies that indicated mFcRn expression in these cells (14). However, while mFcRn is clearly restricted to red pulp MΦs, bFcRn is, in addition, also strongly expressed in the MZ (Figure 3). This MZ display prompted us to perform a more detailed analysis of this compartment. Murine MZ harbors two distinct types of MZ MΦs, of which the metallophilic MΦs within the inner ring are identified by their Sn/CD169 expression, whereas the MZ MΦs located in the outer ring display MΦ receptor with collagenous structure (MARCO) (25). We found that both macrophage subsets display bFcRn in Tg mice. In contrast, no similar expression of mFcRn was detectable in either of these cell types (Figure 3). In peripheral lymph nodes, we observed both mFcRn and bFcRn staining in macrophage-like cells in the interfollicular areas of the lymph node cortex. In addition, we detected bFcRn expression in the CD169-positive, subcapsular sinus (SCS) MΦ, whereas mFcRn was not detected in these cells (Figure 4). These expressional patterns did not change after immunization (data not shown) and largely corroborate the previously reported analysis of the mFcRn expression in the spleen and lymph node (14) with the exception that we did not detect mFcRn in the MZ MΦ in the spleen or in SCS MΦ in lymph nodes of wt animals. These differences in staining pattern may perhaps be attributed to different protocols or reagents.

**Figure 5:**
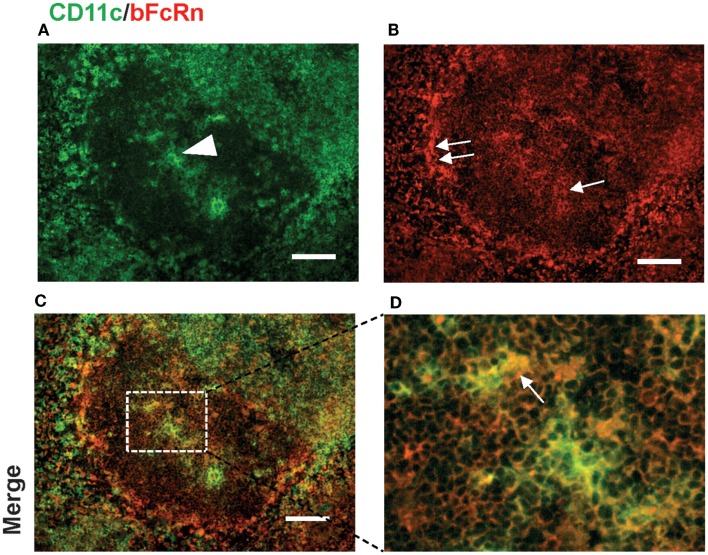
**Expression of bFcRn in splenic dendritic cells in the T cell zone of bFcRn Tg mice**. DCs expressing CD11c (green) within the white pulp [arrowhead in **(A)**] show similar level of bFcRn expression as red pulp MΦs but less intense display of bFcRn [single arrow in **(B)**] compared to marginal zone expression [double arrows in **(B)**], the rectangle delineated by dotted line in inset **(C)** corresponds to **(D)** (representative examples for a cohort *n* > 6, bar size: 100 μm).

We could detect bFcRn expression in CD11c^+^ DCs within the T cell zone, the periarteriolar lymphoid sheath of the white pulp (Figure 5), and this expressional pattern did not change after immunization (data not shown). We could also see bFcRn positivity in the B cell zone of the spleen of bFcRn Tg mice (Figures 2B,F and 3B,F). Since DCs and B cells have crucial roles in the T-dependent immune response, the findings that they express bFcRn prompted us to confirm these data with flow cytometric analysis.

**Figure 6:**
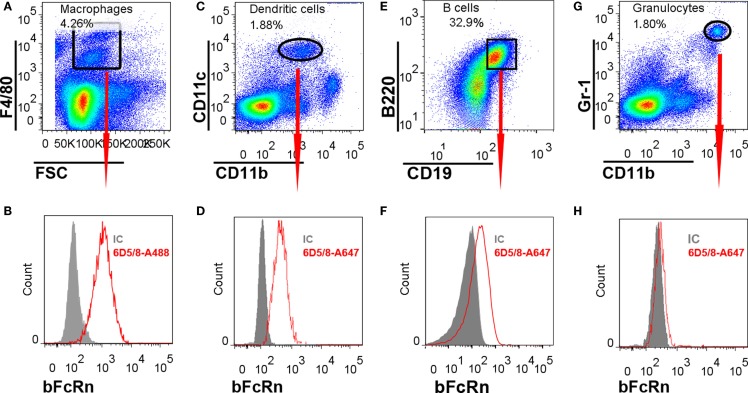
**Flow cytometric analysis of bFcRn expression on splenocyte subsets**. Splenocytes were stained with antibodies recognizing Gr-1, CD11b, CD11c, F4/80, CD19, and B220 markers. For intracellular labeling of bFcRn Alexa Fluor 488 (MΦs) or Alexa Fluor 647 (DCs, B cells, granulocytes), conjugated Z15_6D5/8 mAb were used. Each cell types were gated based on their cell-specific markers **(A,C,E,G)** and their bFcRn expression was presented in related histograms **(B,D,F,H)**. IC, isotype control, 6D5/8: Z15_6D5/8.

We isolated splenocytes from non-immunized mice and found that most of the F4/80^+^ MΦ (Figures 6A,B), CD11b^+^/CD11c^+^ DCs (Figures 6C,D), CD19^+^/B220^+^ B cells (Figures 6E,F), but not the Gr1^+^/CD11b^+^ splenic neutrophil granulocytes (Figures 6G,H) express bFcRn corroborating our immunohistochemical observations. bFcRn expression in MΦs was analyzed with a differently conjugated antibody as compared with the one that we used for DCs, B cells, and granulocytes. Thus, although we could not compare the bFcRn expression in the MΦs to the other cell types, our data suggest that the bFcRn expression in B cells is lower than its expression in DCs. The expression of bFcRn in DCs is another indication that the bFcRn promoter ensures appropriate cell-specific expression as the endogenous mFcRn expression has been detected in these APCs earlier (15–17). It also corroborates our previous result that demonstrated bFcRn expression in bone marrow-derived DCs from the Tg mice using Western blot and Q-PCR analyses (13). On the other hand, the same study did not show bFcRn expression in Tg splenic B cells, in Western blot analysis using a different antibody, which is perhaps less specific for the bFcRn (13). Nevertheless, earlier studies demonstrated mFcRn expression in mouse primary B cells (17, 18) and that has been also indicated in gene expression analysis in immune cells (26). Therefore, bFcRn expression in splenic B cells does not reflect ectopic expression of this transgene in the bFcRn Tg mice.

### Overexpression of bFcRn further increases the frequency of Ag-specific, activated T helper cells in the spleen after immunization

**Figure 7:**
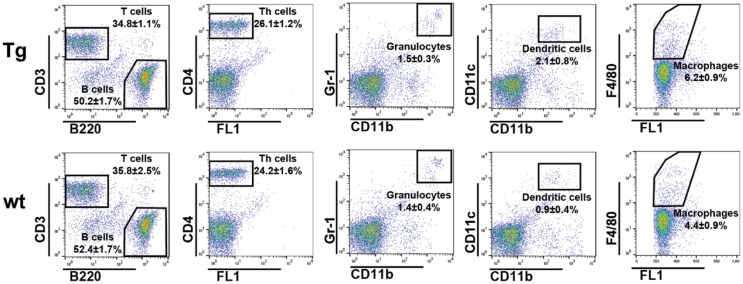
**Flow cytometric analysis of the spleen cell type distribution in non-immunized bFcRn Tg and wt mice**. Splenocytes from non-immunized wt and bFcRn Tg mice (five in both groups) were stained using the following markers: B220, CD3, and CD4 for B-, T-, and helper T cells, respectively, Gr1 and CD11b for neutrophil granulocytes, CD11b and CD11c for dendritic cells, and F4/80 for macrophages. Values on the dot plot panels shown are the mean ± SD.

We previously demonstrated that Ag–IgG IC loaded, bFcRn-overexpressing bone marrow-derived dendritic cells improve antigen presentation significantly as measured by the *in vitro* proliferation of Ag-specific CD4^+^ T cells (13). In order to confirm that bFcRn overexpression leads to increased number of activated Ag-specific helper T cells *in vivo* in the secondary lymphoid organ, we intraperitoneally immunized Tg and wt mice with OVA. This priming was followed by a booster after 14 days, and 7 days later, we isolated splenocytes, reactivated them *ex vivo* with the same Ag (OVA) or with an unrelated Ag (KLH). The number of activated Ag-specific CD4^+^ T cells were determined by IL-2 ELISPOT assay, following previously published protocols (27, 28). Size of the spleens, total numbers of splenocytes, and the ratios and absolute numbers of CD4^+^ T cells, neutrophil granulocytes, DCs, MΦ, B-, and T cells were also compared between non-immunized and immunized Tg and wt mice. In non-immunized mice, we did not observe difference in the ratios of T- and B cells, CD4^+^ T cells, neutrophil granulocytes, DCs, and MΦs (Figure 7), or in the spleen size and number of splenocytes (data not shown) between Tg and wt mice, confirming our previous observation (9).

**Figure 8:**
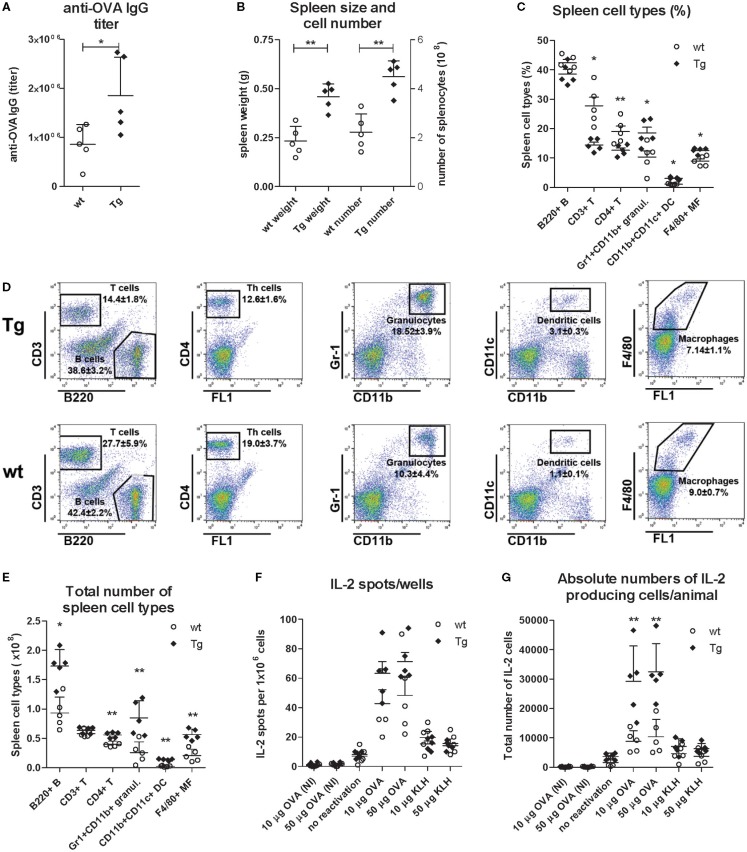
**Comparison of the spleen cell type distribution and the number of IL-2 producer helper T cells in immunized wt and bFcRn Tg mice**. bFcRn Tg and wt mice (five in both groups) were immunized with OVA on days 0 and 14. Mice were sacrificed on day 21 and their immune response were compared by evaluating their OVA-specific IgG titers on day 21 **(A)**, spleen weight and spleen cell numbers **(B)**, distribution of spleen cell types using the following markers: B220, CD3, and CD4 for B-, T-, and helper T cells, respectively, Gr1 and CD11b for neutrophil granulocytes, CD11b and CD11c for dendritic cells, and F4/80 for MΦs **(C–D)**. Total number of spleen cell types was calculated based on the total number of splenocytes and the ratio of the analyzed cell populations **(E)**. IL-2 producer T helper cells of unseparated splenocytes (1 × 10^6^ cells/well) were also analyzed using 10 or 50 μg/ml OVA or non-specific antigen (KLH) for recall activation. As negative controls, splenocytes from non-immunized (NI) mice were stimulated with OVA, or splenocytes from OVA immunized mice were incubated without reactivating them with OVA, or splenocytes from OVA immunized mice were incubated with unrelated antigen (KLH). Data points represent the average spot numbers/ELISPOT wells **(F)**. Total numbers of IL-2 producer helper T cells were calculated based on the total number of CD4^+^ helper T cells in wt and Tg spleen **(G)**. Data on dot plot panels and graphs show the mean ± SD (*P < 0.05; **P < 0.01).

Immunization resulted in an augmented humoral immune response of the bFcRn Tg mice that was reflected by significantly higher OVA-specific IgG titers, larger spleens and increased number of splenocytes on day 21 as compared with their wt controls (Figures 8A,B). We observed significantly higher ratio of T lymphocytes (CD3^+^), helper T (CD4^+^) lymphocytes in wt mice than that of Tg mice, while ratios of neutrophil granulocytes (Gr1^+^/CD11b^+^), DCs (CD11b^+^/CD11c^+^) and MΦs (F4/80^+^) were significantly higher in the spleen of Tg mice than those of wt mice (Figures 8C,D). We also calculated the total numbers of these cell populations based on the total number of splenocytes and their ratios, and observed significantly more B cells, T helper cells, neutrophil granulocytes, DCs and MΦs in the spleen of Tg mice (Figure 8E), reflecting to our previous observations (9, 13). Although Tg spleen contained slightly higher total numbers of CD3^+^ T lymphocytes, this difference was not significant. We also noted that T helper cells represented app. 85 or 70% of the total T cells in the Tg and wt spleen, respectively, suggesting increased activation and proliferation of this cell population in Tg mice (Figure 8E).

Then, we analyzed the number of IL-2 producer T helper cells in unseparated 1 × 10^6^ and 5 × 10^5^ splenocytes from non-immunized and OVA immunized mice. When we counted the IL-2 spots in the ELISPOT wells, we did not observe IL-2 positivity in cases of non-immunized (NI) mice, and found only a few spots when the mice were immunized with OVA but the splenocytes were not reactivated with this Ag. We detected more IL-2 spots when mice were immunized with OVA, and their splenocytes were reactivated with another Ag (KLH). Finally, the highest number of spots appeared when mice were immunized with OVA, and their splenocytes were reactivated with the same Ag (Figure 8F). (We got similar trends with fewer spots when 5 × 10^5^ splenocytes were used; data not shown.) Although these data did not show significant difference between Tg and wt mice, they reflected augmented T helper cell activation in Tg mice as there were significantly fewer T helper cells in the wells of Tg mice compared to their controls (Figure 8C). In addition, immunization of Tg mice resulted in larger spleen and more splenocytes and calculating all these factors clearly indicated that the spleen of the immunized Tg mice contained approximately threefold more activated, Ag-specific T helper cells as compared to their wt controls (Figure 8G). These data validated our previous *in vitro* antigen presentation assay using bone marrow-derived DCs (13).

### Overexpression of bFcRn expands the number and size of germinal centers

To determine whether increased number of Ag-specific helper T cells in bFcRn Tg mice can be correlated with GC formation, GCs were assessed by quantitative, cross-sectional analysis in spleens of TNP-KLH-challenged Tg and wt mice during the secondary immune response at different time points after the booster immunization in which we observed significant difference in the humoral immune response comparing the bFcRn Tg mice and their wt controls (29).

**Figure 9:**
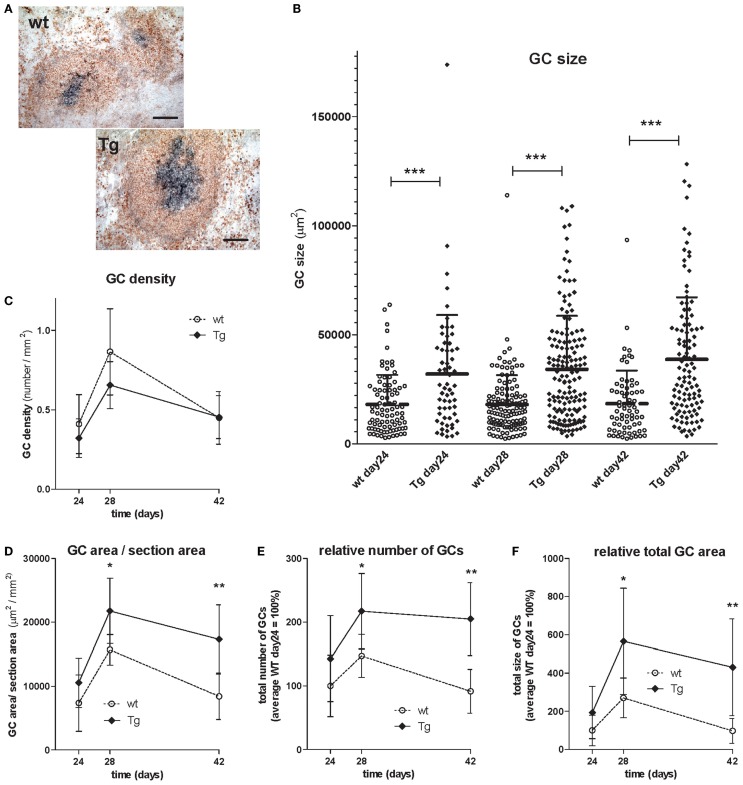
**Analysis and comparison of germinal centers (GC) of TNP-KLH immunized wt and bFcRn Tg mice after booster immunization on day 21**. bFcRn Tg and wt mice were immunized with KLH-TNP, boosted 3 weeks later and GCs were analyzed on day 24, 28, and 42 of the immune response (3 Tg and wt mice in each group). **(A)** Well-established GCs were identified with their IgD (naïve B cells in red) and PNA (GC B cells in blue) positivity (representative examples for a cohort *n* > 6; scale bar: 100 μm). **(B)** Each dot represents an individual GC and the average size of the GCs is approximately twofold in the bFcRn Tg mice. Lines of the scatter dot plot indicates means with SDs; the high SD values indicate a non-normal, right-skewed distribution of GC sizes, as the result of a small frequency of very large GCs density of GCs in the spleens [number of GCs in area units (square millimeter) of spleen sections] **(C)**, average ratio of the GC size per section area **(D)**, relative number of GCs **(E)**, and total GC area **(F)** in the spleens were evaluated. Inserts **(E,F)** represents data relative to the average wt data on day 24 of immunization. Data show the mean ± SD (*P < 0.05; **P < 0.01; ***P < 0.001).

Similarly to the OVA immunization experiment, we found that TNP-KLH immunization resulted in larger spleen and higher Ag-specific IgG titers in the Tg mice as compared with their controls (data not shown). Cross-sectional GC size in spleens were evaluated by quantitative analysis at different time points after booster immunization as we focused on the secondary immune response and analyzed the GCs after the booster immunization (day 21). We evaluated 606 GCs in total of 36 sections prepared on days 24, 28, and 42 of immunization from three Tg and wt mice at each time point (Table 2). Ag-specific B cells within GCs were identified by PNA staining, while surrounding non-GC B cells were counterstained by their IgD positivity (Figure 9A). GCs varied widely in size from a minimum of 2299 μm^2^ to a maximum of 113,986 μm^2^ in case of the wt animals, or from a minimum of 3427 μm^2^ to a maximum of 173,799 μm^2^ in case of the Tg mice with a wider range of distribution in the Tg mice. These data gave the median values of 14,431 μm^2^ for wt and 26616 μm^2^ for Tg on day 24, 15,122 μm^2^ for wt and 29,655 μm^2^ for Tg mice on day 28, and 14,972 μm^2^ for wt and 30,263 μm^2^ for Tg mice on day 42 indicating that the average size of the GCs is approximately twofold in the bFcRn Tg mice. Distribution of GC sizes were non-normal, right-skewed, indicating that most of the GCs were small and medium sizes but some of them were extremely large, especially in the Tg spleens (Figure 9B). GC density [number of GCs in area units (square millimeter) of spleen sections] did not differ between wt and Tg groups, in both cases it increased radically after the booster immunization (day 21), peaked on day 28, and then gradually reduced (Figure 9C). From day 28 of immunization, we observed larger average ratio of the GC size per section area (Figure 9D), many more GCs (Figure 9E), and much larger total GC area (Figure 9F) in Tg spleen comparing with those of wt.

**Table 2:** **Survey of cross-sectional GC size evaluation (number of GCs analyzed)**.

No. of evaluated GCs														

Day^a^	wt1^b^		wt2^b^		wt3^b^		Tg1^b^		Tg2^b^		Tg3^b^			
	S1	S2	S1	S2	S1	S2	S1	S2	S1	S2	S1	S2	*wt* Σ*^c^*	*Tg* Σ^c^
24	26	17	1	12	13	19	4	10	11	20	6	10	88	61
28	14	12	28	20	20	30	23	38	29	38	10	16	124	154
42	19	12	5	13	4	16	11	17	16	37	9	20	69	110
													281	325

*^a^wt and Tg mice were immunized on days 0 and 21, and their spleen was analyzed at the indicated time points*.

*^b^Number of GCs evaluated per two independent spleen sections per mice (S1 and S2, distance at least 500 μm). Three wt and three Tg mice were analyzed at each time point*.

*^c^Total number of GCs evaluated per time point in wt or Tg group*.

### Tg mice demonstrated efficient immune response even to very low dose of Ag

**Figure 10:**
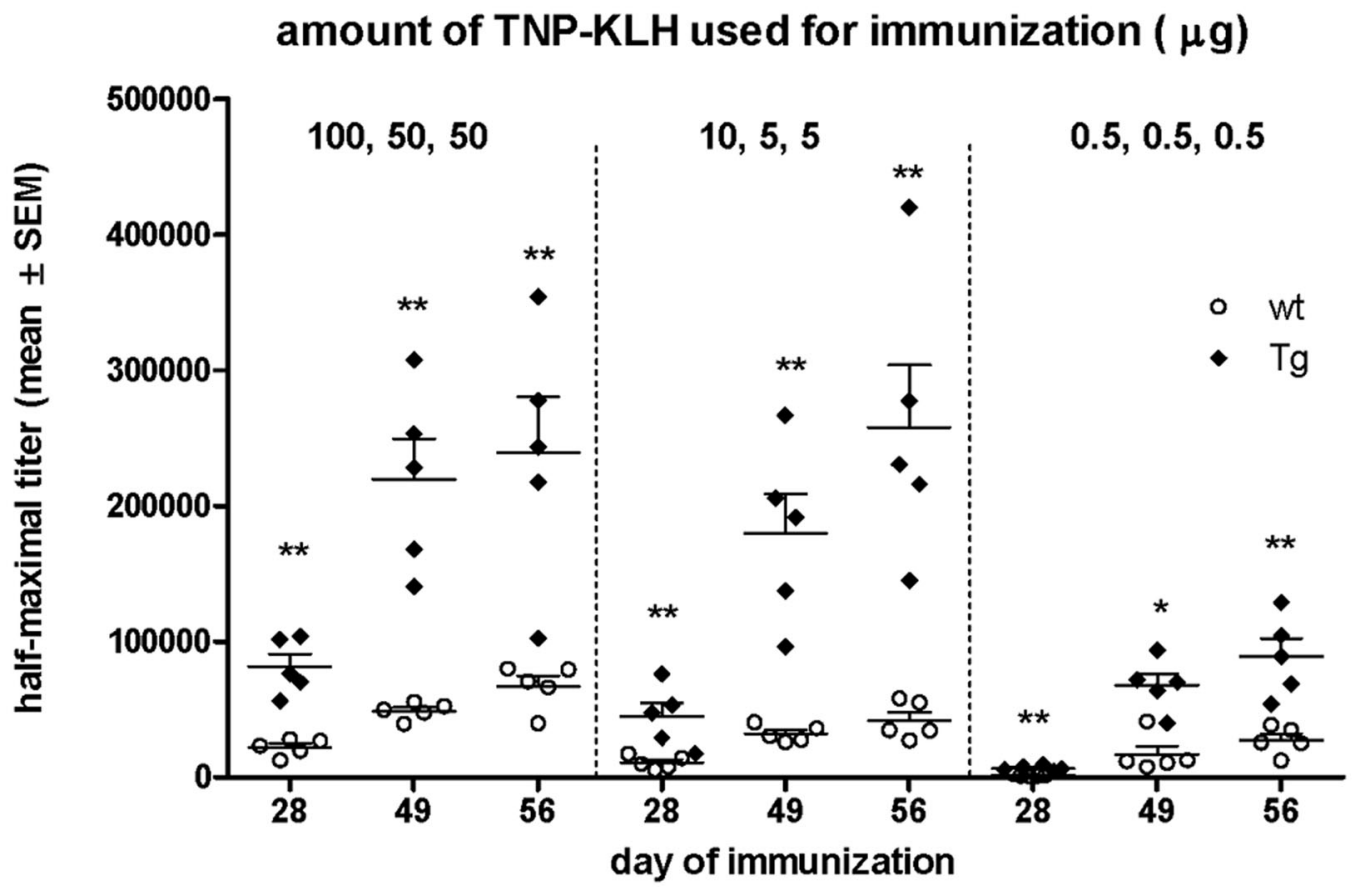
**Comparison of the Ag-specific IgG titer in wt and bFcRn Tg mice when low amount of Ag was used for immunization**. Three cohorts of Tg and wt mice were i.p. immunized with different amount of TNP-KLH (see also Table 1) and their TNP-specific IgG titers were measured by ELISA. Data show the mean ± SD (*P < 0.05; **P < 0.01).

We also wanted to test whether overexpression of bFcRn has an impact on the dose-dependency of the immune response *in vivo*. Therefore, we immunized bFcRn Tg and wt mice with different amounts of TNP-KLH in three cohorts, based on the immunization protocols summarized in Table 1. As readout, we analyzed the TNP-specific serum titers. As expected from our previous studies, Tg mice produced four- to sixfold higher TNP-specific titers with even higher maximum titers, wider titer range as compared with their wt controls. Surprisingly, even the medium antigen dose used for cohort #2 (10/5/5 μg/mouse) has led only in Tg mice already to an optimal TNP-specific humoral immune response, as immunization with higher antigen doses (cohort #1) did not result in significantly higher titers. In contrast, in wt mice, even the highest antigen doses did not result in titers equivalent to those of Tg mice. Even more important is the finding that Tg mice are very responsive to tiny amounts of antigen as the Ag doses used in cohort #3 (0.5/0.5/0.5 μg/mouse) triggered a significant humoral immune response (Figure 10).

## Discussion

In this study, our objective was to gain further insights into the augmented humoral immune response of the bFcRn Tg mice, which we observed using T-dependent Ags (8, 9, 12, 13). First, we analyzed the microarchitecture of the bFcRn Tg spleen and found normal T-, B cell, and MZ compartmentalization indistinguishable from wt controls (Figure 1). Then, we compared the transgenic bFcRn expression to the endogenous mFcRn expression in the spleen and lymph node. Our mFcRn expression analysis in these secondary lymphoid organs largely corroborated with previous observation (14) with the exception that we could not detect mFcRn expression in the MZ MΦs in the spleen or in the SCS MΦs in the lymph node. On the other hand, we detected bFcRn expression in the outer and inner rings of the MZ, representing the MARCO^+^ MZ MΦs and the CD169^+^ marginal metallophilic MΦs, in addition to the mFcRn-positive components in the red pulp. Importantly, these findings indicate that the lineage-specific regulation of bFcRn expression is similar to its murine ortholog, although Tg bFcRn has a stronger expression in the MZ MΦs compared with red pulp MΦs (Figures 2–4). This enhanced expression of bFcRn by MZ MΦs in spleen and SCS MΦs in peripheral lymph nodes may provide augmented stimulation for the humoral immune response. It was previously shown that MΦs of the splenic MZ are dispensable for T cell activation (30); however, a recent study demonstrated their necessity for progression of T-dependent B cell responses and formation of GCs, in a fashion analogous to lymph node MΦs (31). Indeed, the position and phenotype of CD169^+^ marginal metallophilic MΦs resemble to that of MΦs lying just underneath the SCS of lymph nodes (32). SCS MΦs were shown to be involved in Ag–IgG IC translocation on the surface of these MΦs from their protrusions into the sinus to areas of the MΦs in contact with follicular B cells. At the interface between these MΦs and follicular B cells, there is accumulation of native Ag or ICs leading to cognate B cell activation. Alternatively, in case of non-cognate B cells, ICs bind to the complement receptor on B cells and then transported into the follicle, facilitating deposition of these ICs on the follicular dendritic cells (FDCs) (33–35). More recently, it was demonstrated that these SCS MΦs are poorly endocytic, have low degradative capacity, and retain ICs on the cell surface. Such function is considered as a specialized antigen-presenting cell function, and that these ICs contribute to GC formation and affinity maturation (36). Yet, these authors did not exclude the possibility that ICs are transported inside these cells in a specialized form of transcytosis. Based on our data that demonstrates strong bFcRn expression in marginal metallophilic MΦs in the spleen or SCS MΦs in lymph nodes, it is tempting to hypothesize that the bFcRn expression in these cells may contribute to IC protection and transportation leading to increased B cell activation, coupled with more efficient GC formation and affinity maturation. Further analyses tracing the transport of detectable antigen in immune complex form may provide further understanding in this immune complex transport pathway. The role of MARCO^+^ MZ MΦs in capturing ICs and stimulating B cells, including MZ B cells is less characterized, but we cannot exclude the possibility that this process is also mediated by FcRn. Further studies are warranted to carefully analyze the expression of mFcRn and hFcRn in these cells and to validate this hypothesis.

In parallel with follicular B cell stimulation, the activation of T helper cells by professional APCs represents the other arm of the T-dependent humoral immune response. After the initial contact with antigen, B cells travel to the border between the T cell zone and B cell follicle where they interact with cognate T cells to form Ag-specific cell clusters. Thus, activated T helper cells are crucially important in stimulating cognate B cells and consequently strongly influence Ag-driven B cell activation and then their maturation within the GCs after some of these activated cognate T helper cells become follicular T helper (T_FH_) cells. FcRn plays major roles in antigen presentation of Ag–IgG IC by professional APCs and in generating Ag-specific antibodies (15, 17, 37–39). This process is initiated at the cell surface of most professional APCs, as Ag-IgG IC binds to the FcγRs which triggers both the internalization of this IC and its delivery into endocytic vesicles. While FcRn recycles monomeric IgG from these compartments onto the cell surface, it directs the uptaken Ag-IgG IC into vesicles where processing of the complexed antigen releases epitopes that are loaded onto MHC class I (MHC-I) and class II (MHC-II) molecules that subsequently stimulate the activation of cognate CD8^+^ and CD4^+^ T cells, as has recently been reviewed (5).

We have previously demonstrated that bone marrow-derived dendritic cells isolated from the bFcRn Tg mice stimulate CD4^+^ T cell proliferation much more efficiently when loaded with Ag-IgG ICs than those of wt controls (13). Our current findings that splenic DCs of Tg mice express bFcRn (Figures 5 and 6) supports that previous observation and suggests that these cells have an augmented APC activity in the spleen on the course of the immune response. This assumption was confirmed by showing that immunization results in threefold more activated, Ag-specific helper T-cells in the Tg spleen than that of wt controls (Figure 8). Immunization resulted not only more helper T cells in the Tg spleen but substantial differences in cell populations (Figure 8), although there were no such differences between non-immunized Tg and wt mice (Figure 7) supporting our previous observations (9, 12).

This remarkable difference in the splenic T helper cell activity of the Tg mice urged us to analyze the size distributions and kinetics of the formation of GCs in the spleen during the secondary immune response. We observed that most of the GCs were small and medium sizes in the wt spleen (Figure 9) similarly as described before (40). In the case of Tg mice though, while there were many small or medium sized GCs in the TG spleen, many of them were much larger than the controls and thus Tg GCs were averagely twice as large as that of the controls on days 28 and 42 of the immunization. Although, we found similar GC density both in wt and Tg spleen, based on the larger spleen in Tg mice the total number of GCs were almost doubled in the Tg mice than that of wt controls (Figure 9). These observations confirmed our previous studies that showed many more antigen-specific B cells in the bFcRn Tg mice than their wt controls (8, 9).

The much larger size of the Tg GCs could be the result of the many more activated T helper cells, and we cannot exclude the possibility that the strong bFcRn expression in the MZ MΦs contribute to this outcome, too, resulting in more cognate B cell activation, and more Ag-IgG ICs into the GCs (as discussed earlier).

Furthermore, our data show that primary splenic B cells from the Tg mice express bFcRn, too, although at lower expressional level than DCs (Figures 2 and 6). This new finding corroborate to previous studies that demonstrated mFcRn expression in mouse splenic B cells (17, 18), although other studies failed to show FcRn expression in primary B cells or B cell lines (38, 41, 42). The expression of mFcRn in splenic B cells has been also indicated by the Immunological Genome Project database (26), and thus these studies support our hypothesis that the B cell specificity of the transgenic bFcRn expression reflects to the endogenous mFcRn, too. The role of FcRn in B cell has been hypothesized to regulate IgG (and albumin) catabolism and involve in APC function of the B cells (17, 18). The potential role of FcRn in B cell APC function is an exciting hypothesis as activated B cells present antigens to cognate helper T cells in the initial phase of the immune response and later to cognate T_FH_ cells during GC center reaction. Particularly in the GC reaction, B cells recognizing Ag–IgG ICs presented by FDCs receive simultaneous activating signals from their B cell receptor (BCR) and inhibitory signals via cross-linking of the inhibitory FcγRIIB, which thereby sets a threshold for B cell activation (43). As a consequence, B cells that lose their affinity for the presented Ag will only receive signals through the inhibitory FcγRIIB, which could result in apoptosis, while B cells carrying a BCR with sufficiently high affinity will become fully activated and proceed further to antibody production (43). Via this route, these activated GC B cells uptake Ag–IgG IC via robust BCR-mediated Ag capture from FDCs (and thus differently compared to, e.g., DCs, which internalize all kinds of Ag–IgG ICs via their classical Fcγ receptors) and present the processed Ag on MHC complexes to T_FH_ cells. Higher BCR affinity is directly associated with greater Ag capture and uptake leading to a higher density of peptide–MHC presentation on the surface of the B cell. As a consequence, these B cells have stronger interaction with the T_FH_ cells, which then results in greater magnitude of T cell help for re-entry into the dark zone where these B cells undergo somatic hypermutation and can collect more affinity-enhancing mutations (44). From the perspective of our study, we hypothesize that FcRn may also be involved in this APC activity of GC B cells, and the overexpression of the bFcRn in these B cells may result in better T_FH_ activation and thus increased B cell proliferation explaining the larger GCs that we observed in Tg spleen (Figure 9). The appropriate affinity maturation in Tg animals that overexpress FcRn has been demonstrated earlier, indicating that the affinity of the Ag-specific IgG was at least as good in Tg mice as in the wt controls (9). Furthermore, our recent antibody development projects resulted in very high-affinity mAbs from our bFcRn Tg mice (unpublished observation) and also from our FcRn Tg rabbits that have similar phenotype to the bFcRn Tg mice (11). Thus, we believe that FcRn overexpression improves affinity maturation and analyzing this important aspect more systematically is one of our most important goals.

Another potential outcome of the larger and many more GCs is that the Tg mice may develop increased diversity of Ag-specific antibodies. It was previously demonstrated that the number of activated T helper cells strongly influences the number of B cell clones that seeds GCs (45). Another aspect of the competition between activated B cells is that B cells that have higher Ag-affinity BCR uptake and present more Ag-specific peptide–MHC class II complex to cognate T helper cells than those that have lower Ag-affinity BCR when GC formation is initiated. Since there are generally limited number of cognate T helper cells, only those B cells receive T helper cell stimulus and seed GCs that have high-affinity BCR. This interclonal competition was detectable over a wide range of Ag doses and it was also shown that increased number of Ag-specific T helper cells lead to an overall increase of high- and low-affinity B cell activation and proliferation indicating that T cell help is indeed limiting at this early stage. This study also revealed that increased T cell help is associated with increased GC occupancy (45). Thus, it is possible that the more activated T helper cells we found in Tg spleen lead to more founder B cells seeding the GCs in the bFcRn Tg mice. In parallel to this, we also detected many more GCs in the Tg spleen that have been formed potentially from different founder B cells. These data suggest greater diversity of Ag-specific B cells in the bFcRn Tg mice supporting our previous observation, albeit we found increased diversity at IgM, but not at IgG level in that study (13). We have initiated antibody sequencing and epitope binning protocols to analyze this issue more extensively and gain further information about the efficiency of the affinity maturation, too, in the bFcRn Tg mice.

We have already demonstrated that these bFcRn Tg mice could effectively induce immune response against weakly immunogenic antigens (12). Another important advantage would be for many antibody development protocols to generate mAbs at a point in time when only tiny amount of purified antigen is available. As referred above, the number of activated founder B cells that seed GCs depends primarily on the number of cognate T helper cells and less on the amount of Ag (45), although very low dose of antigen frequently results in non-responsiveness. To analyze this relatedness, we immunized Tg and wt mice with high, medium, and very low amount of TNP-KLH and analyzed their efficiency to develop Ag-specific immune responses. As expected, we found that the bFcRn Tg mice produced significantly higher level of Ag-specific IgG titers, and their superior immune response was demonstrated by their immune response capability to produce antibodies even when very low amount of Ag (three times 0.5 μg per mouse) was used (Figure 10). This could be advantageous to develop antibodies against of recombinant proteins or other unique antigens that are difficult to produce.

We concluded that immunization results in multifold-more activated, Ag-specific T helper cells, and significantly larger and more GCs in the spleen, and the ability to raise Abs against very low dose of Ag in bFcRn Tg mice compared with their wt controls. These findings suggest the advantage of these Tg mice for antibody development against weakly immunogenic targets (e.g., GPCR, ion channels) and also for broader epitope recognition.

## Conflict of Interest Statement

Attila Végh, Judit Cervenak, Péter Károly Jani, Imre Kacskovics, and István Kurucz are employees of ImmunoGenes using patented, genetically modified animal.that overexpress FcRn for the production of polyclonal and monoclonal antibodies. The remaining authors have no conflict of interest to declare.

## Abbreviations

Ag, antigen; FcRn, neonatal Fc receptor; GC, germinal center; KLH, keyhole limpet hemocyanin; mAb, monoclonal antibody; MZ, marginal zone; Tg, transgenic, wt, wild type.
